# Emerging infectious diseases in Brazil.

**DOI:** 10.3201/eid0402.980234

**Published:** 1998

**Authors:** L J da Sliva


3. Lin J, Smith MP, Chapin KC, Baik HS, Bennett GN, Foster JW. Mechanisms of acid resistance in enterohemorrhagic *Escherichia coli*. Appl Environ Microbiol 1996;62:3094-100.
4. Michino H, Araki K, Minami S, Takaya S, Sakai N. Investigation of large-scale outbreak of *Escherichia coli* O157:H7 infection among schoolchildren in Sakai City, 1996. In: Proceedings of the 32nd Joint Conference on Cholera and Related Diarrheal Diseases, U.S.-Japan Cooperative Medical Science Program(USJCMSP); 14-16 Nov 1996; Nagasaki University, Nagasaki, Japan. USJCMSP: 1996. p. 84-9.
5. Nathan R. American seeds suspected in Japanese food poisoning epidemic. Nat Med 1997;3:705-6.

### Irradiation Pasteurization of Solid Foods

**To the Editor:** Osterholm and Potter have made a strong case for irradiation pasteurization of solid foods that enter kitchens as raw agricultural commodities, such as meat, poultry, and seafood (1). Irradiation pasteurization was advocated to protect against foodborne diseases caused by common pathogens such as *Campylobacter*, *Cryptosporidium*, *Escherichia coli*, *Listeria*, *Salmonella*, and *Toxoplasma* (2). An additional rationale for irradiation pasteurization is bacterial resistance to antimicrobial drugs, a major health concern, which will undoubtedly increase in magnitude unless new approaches become available (3). The widespread use of antibiotics in animal husbandry may be the cause of some of this resistance, for example, in vancomycin-resistant enterococci associated with the agricultural use of glycopeptide antibiotics (4,5). Furthermore, resistance to glycopeptide antibitiotics can be transferred from enterococci to other gram-positive organisms, at least in the laboratory (6). Thus, resistant bacterial strains from animal sources may enter the human population through contaminated food without necessarily causing immediate disease but resulting in expanded human reservoirs of antimicrobial resistance through horizontal gene transfer (7). When such bacterial strains are subsequently transmitted to a susceptible person, serious disease could result, which would be exceedingly difficult to treat (8). Irradiation pasteurization of solid foods could reduce the magnitude of transfer of resistance genes through contaminated foods.

**Stephen Moses and Robert C. Brunham**
University of Manitoba, Winnipeg, Canada

### References

1. Osterholm MT, Potter ME. Irradiation pasteurization of solid foods: taking food safety to the next level. Emerg Infect Dis 1997;3:575-6.
2. Monk JD, Beuchat LR, Doyle MP. Irradiation inactivation of food-borne microorganisms. Journal of Food Protection 1995;58:197-208.
3. Gold HS, Moellering RC. Antimicrobial-drug resistance. N Engl J Med 1996;335:1445-53.
4. Bates J, Jordens JZ, Griffiths DT. Farm animals as a putative reservoir for vancomycin-resistant enterococcal infection in man. J Antimicrob Chemother 1994;34:507-14.
5. Van de Bogaard AE, Jensen LB, Stobberingh EE. Vancomycin-resistant enterococci in turkeys and farmers [letter]. N Engl J Med 1997;337:1558-9.
6. Leclerq R, Derlot E, Weber M, Duval J, Courvalin P. Transferable vancomycin and teicoplanin resistance in *Enterococcus faecium*. Antimicrob Agents Chemother 1989;33:10-5.
7. Davies J. Inactivation of antibiotics and the dissemination of resistance genes. Science 1994;264:375-82.
8. Swartz MN. Hospital-acquired infections: diseases with increasingly limited therapies. Proc Natl Acad Sci U S A 1994;91:2420-7.

### Emerging Infectious Diseases in Brazil

**To the Editor:** Hooman Momen's update on emerging infectious diseases in Brazil (1) appears to be based solely on notifiable disease data, which cannot adequately describe the current situation. Additional data in several areas may be useful.

Parasitic diseases: Dr. Momen's update restricts itself to protozoal diseases and does not distinguish between mucocutaneous and visceral leishmaniasis. Visceral leishmaniasis is in fact expanding in many suburban and urban areas in the northeast. Mucocutaneous leishmaniasis, after a small retreat following extensive deforestation, has made a comeback; and in many suburban areas in Rio de Janeiro and São Paulo, in the southeast, transmission is occurring, probably because of changes in sandfly ecology (1).

A helminthic disease of interest is mansoni schistosomiasis, which has been expanding its area of transmission, reaching over to Santa Catarina, in the south, to Pará in the north, expanding also westward, to Mato Grosso and Mato Grosso do Sul. The number of cases, as well as the associated illness, has possibly been reduced, but there is no doubt that the disease can be found in a much larger area than 20 years ago. Other emerging helminthiases of interest, albeit not of public health concern, are onchocerciasis, still restricted to the Yanomami group in Roraima, bordering Venezuela; *Angiostrongylus costaricensis* infection (2), found in the south, Rio Grande do Sul; and some cases of lagochilascariasis, reported from Pará.

Viral diseases: As Dr. Momen pointed out, dengue is by far the most serious emerging viral disease in Brazil, and the area occupied by *Aedes aegypti* is expanding. Dengue hemorrhagic fever has occurred occasionally, but no outbreaks have been recorded. However, measles is no longer a problem; the outbreaks have been controlled.

There is no evidence to support that hepatitis B is declining because of vaccination. Vaccination is still restricted to areas of high prevalence. Other states are beginning vaccination programs in newborns, but it will be some time before these programs have any effect on prevalence. As to hepatitis C, because diagnostic testing is only recently becoming widespread, we are probably experiencing an increase in detection rather than in incidence.

Other notable agents are Mayaro and Oropouche viruses, which are arthropod-borne and among the most common causes of febrile illness in the Amazon region. *Aedes albopictus*, found all over the country, could be a potential vector (3). Apart from HIV, other retroviruses are cause for concern: HTLV-I and HTLV-II screening is recommended for blood banks, and enough data exist to conclude that the infection is widespread in the country but with a low prevalence (0.4% and 0.1%, respectively). Clusters of disease have not been identified, but adult T-cell leukemia/lymphoma is far from a curiosity (4).

Bacterial diseases: Brazilian purpuric fever, caused by *Haemophilus influenzae* biogroup aegyptius, was first reported in outbreaks in the central-south part of the country (western São Paulo, eastern Mato Grosso do Sul, and northwestern Paraná) about 10 years ago, causing a syndrome much like meningococcemia (5). For enteric infections, the limited data available present interesting trends. *Salmonella* Enteritidis is rising and *S*. Typhimurium is declining in São Paulo and the southern states. These trends may reflect improved sanitation and increased use of industrialized foods and contaminated animal feeds (6).

Fungal diseases are not reportable, but many epidemiologic studies have been conducted. Paracoccidioidomycosis (South American blastomycosis) was unheard of in the Amazon region, never being found in native habitants; however, because of environmental and socioeconomic changes, the infection is now being identified (7).

Antimicrobial resistance is a serious problem, not only within hospitals, but also in the community. Penicillin-resistant pneumococcus is not yet a widespread problem, but it has been detected (8); the same situation exists with regard to *Mycobacterium tuberculosis* (9).

The problem of emerging infectious disease is gaining increasing attention in Brazil, and published reports together with notifiable disease data underline the main points of concern.

**Luiz Jacintho da Silva**
Clinica Medica, FCM, Unicamp, Campinas, Brazil

### References

1. Gomes AM. Sandfly ecology in the State of São Paulo. Mem Inst Oswaldo Cruz 1994;89:457-60.
2. Rambo PR, Agostini AA, Graeff-Teixeira C. Abdominal angiostrongylosis in southern Brazil—prevalence and parasitic burden in mollusk intermediate hosts from eighteen endemic foci. Mem Inst Oswaldo Cruz 1997;92:9-14.
3. Smith GC, Francy DB. Laboratory studies of a Brazilian strain of *Aedes albopictus* as a potential vector of Mayaro and Oropouche viruses. J Am Mosq Control Assoc 1991;7:89-93.
4. Farias de Carvalho SM, Pombo de Oliveira MS, Thuler LC, Rios M, Coelho RC, Rubim LC, et al. HTLV-I and HTLV-II infections in hematologic disorder patients, cancer patients, and healthy individuals from Rio de Janeiro, Brazil. J Aquir Immune Defic Syndr Hum Retrovirol 1997;15:238-42.
5. The Brazilian Purpuric Fever Study Group. Brazilian purpuric fever identified in a new region of Brazil. J Infect Dis 1992;165:S16-9.
6. Tavechio AT, Fernandes SA, Neves BC, Dias AM, Irino K. Changing patterns of Salmonella serovars: increase of *Salmonella* Enteritidis in São Paulo, Brazil. Rev Inst Med Trop São Paulo 1996;38:315-22.
7. Coimbra Jr CE, Wanke B, Santos RV, do Valle AC, Costa RL, Zancope-Oliveira RM. Paracoccidiodin and histoplasmin sensitivity in Tupi-Monde Amerindian populations from Brazilian Amazonia. Ann Trop Med Parasitol 1994;88:197-207.
8. Brandileone MC, Vieira VS, Casagrande ST, Zanella RC, Guerra ML, Bokermann S, et al. Prevalence of serotypes and antimicrobial resistance of *Streptococcus pneumoniae* strains isolated from Brazilian children with invasive infections Pneumococcal Study Group in Brazil for the SIREVA project. Regional System for Vaccines in Latin America. Microb Drug Resist 1997;3:141-6.
9. Pinto WP, Hadad DJ, Palhares MC, Ferrazoli L, Telles MA, Ueki SY, et al. Drug resistance of *M.tuberculosis* isolated from patients with HIV infection seen at an AIDS Reference Center in São Paulo, Brazil. Rev Inst Med Trop São Paulo 1996;38:15-21.

